# How strenuous is esports? Perceived physical exertion and physical state during competitive video gaming

**DOI:** 10.3389/fspor.2024.1370485

**Published:** 2024-07-10

**Authors:** Chuck Tholl, Markus Soffner, Ingo Froböse

**Affiliations:** ^1^ Department of Movement-Oriented Prevention and Rehabilitation Sciences, Institute of Movement Therapy and Movement-Oriented Prevention and Rehabilitation, German Sport University Cologne, Cologne, Germany; ^2^ Department of Sports Medicine, University of Wuppertal, Wuppertal, Germany

**Keywords:** video games, RPE, sedentary behavior, ANOVA, fatigue, rest

## Abstract

**Introduction:**

Esports or competitive video gaming is a rapidly growing sector and an integral part of today's (youth) culture. Esports athletes are exposed to a variety of burdens, that can potentially impact an athlete's health and performance. Therefore, it is important that esports athletes are aware of (physical) burden and exertion associated with esports. For this purpose, a study was conducted to evaluate the influence of competitive video gaming on the perceived physical exertion and the perceived physical state (PEPS).

**Methods:**

Thirty-two healthy male esports athletes participated in two competitive video gaming sessions lasting 90–120 min, interrupted by a 10-minute passive sitting break. Repeated measures of perceived physical exertion (Borg Categorial Ratio-10 scale) and perceived physical state were recorded before, during, and after each video game session. Repeated measures ANOVA and Friedman's test were used for statistical analysis.

**Results:**

The results showed a significant difference in all dimensions of the PEPS (*p <* 0.05) as well as in Borg scale (*p <* 0.001). Post-hoc tests revealed significant increases in Borg scale between baseline measurements (T0: 1.0 ± 1.0) and after the first competitive video gaming session (T1: 2.4 ± 1.3, *p *< 0.001), as well as after the second competitive video gaming session (T3: 3.0 ± 1.7, *p* < 0.001). Furthermore, there was a significant reduction in perceived exertion between the measurement time after the first competitive video gaming session (T1) and the break (T2: 1.3 ± 1.2, *p* < 0.001). The PEPS dimensions activation, trained, and mobility showed similar significant changes in post-hoc analysis.

**Discussion:**

The results indicate that the perceived physical burden significantly increases during esports participation. As the duration of competitive video gaming extends, the perceived physical state decreases and perceived physical exertion increases. A passive break between two video game sessions can at least partially restore physical exertion and physical state. However, this break neither returns the scores to their baseline levels nor prevents a further decline in scores during the second video game session. Over time and with a lack of observation, this could result in health and performance limitations.

## 1 Introduction

The video gaming sector has been a rapidly growing area for several years. This has led to a massive increase in video game players, spectators, and the global video game market over the past decade (1). It is estimated that there were 3.4 billion video game players globally in 2023 (1). One part of the video gaming sector is electronic sports (esports), also known as competitive video gaming (2). In esports, athletes compete against each other in different virtual environments. Today, esports athletes compete in tournaments with millions of dollars in prize money, attract millions of viewers and serve as role models, especially for young people (3, 4). Therefore, it is not a short-term trend, but an integral part of today's (youth) culture and competitive sports industry.

With the growing interest in esports, the performance and health of esports athletes has become a focus for organizations and researchers. Esports athletes train between 4 and 10 h/day to develop (game-)specific abilities depending on their skill level and the game genre (5, 6). The requirements range from mechanical skills to control the digital environment, to tactical-cognitive skills to plan moves or cooperate with teammates, to psychological skills such as resilience (7). Currently, there is a lack of evidence in which esports games players spend the most time playing, or which skills require the most training to develop. In addition, esports athletes are exposed to a variety of burdens, that can affect an athlete's health and/or performance (8, 9). Various biopsychosocial stressors such as prolonged sitting (10), high mental stress, or team issues are present in esports (11). A recent systematic review on stress in esports revealed different psychophysiological responses (12). Interestingly, the participation in non-competitive esports games does not seem to be associated with changes. In competitive settings however, mixed results have been found, indicating potential changes in the heart rate, heart rate variability, and blood pressure (12). Research has shown that esports athletes’ perceptions of psychological stress can be influenced by winning or losing their games (13).

In addition to these psychophysiological responses, the physical burdens of esports and its potential consequences have previously been discussed (14). Video gaming and esports by their (current) nature are mostly sedentary behaviors combined physical inactivity (15), monotonous and prolonged sitting (8), and repetitive movements of the upper extremities (16). Except for exercise or virtual reality games, which require physical movements to interact with the digital environment and could increase physical activity (17). As a result, excessive video gaming may lead to the occurrence of musculoskeletal disorders (14). Consequently, not only could the health of esports athletes be compromised, but their performance may also be affected due to impairments. Such physical ailments could lead to early retirement (9). Therefore, it is important that esports athletes are aware of physical burden and exertion in order to counteract these consequences. This requires good self- and body perception. However, there is a lack of evidence on body perception during competitive video gaming. As mentioned above, perceived physical exertion is of particular interest in terms of injury prevention and intensity control. The findings could be useful for load management, intensity control and self-perception in esports.

Therefore, the overall aim of this study is to examine the perceived physical burdens of esports athletes during competitive video gaming. We hypothesized that the perceived physical exertion would increase and the perceived physical state would decrease over time.

## 2 Materials and methods

### 2.1 Study design

**Figure 1:**
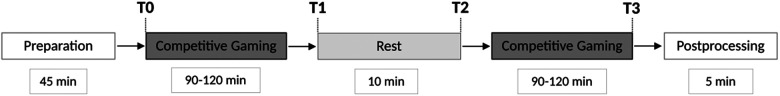
Study design.

This study used a repeated measures, within-group, non-randomized design. Due to the exploratory approach and the non-standardizable nature of the video game activity the study focused on within-group study design. This allows the participants to act as their own control, reducing individual variability for between-group comparisons. The study took place in the laboratory of the Institute of Movement Therapy and Movement-oriented Prevention and Rehabilitation at the German Sport University Cologne. Between 06/2023 and 12/2023 esports athletes were recruited for a five-to-six-hour investigation. The participants took part in two competitive video gaming sessions of 90–120 min interrupted by a 10-minute passive sitting break (Figure 1). At the measurement points (T0-T3) and during video game play, objective and subjective parameters were examined. The study protocol followed the ethical principles defined in the declaration of Helsinki and were approved by the ethical committee of the German Sport University Cologne (reference: 093/2023).

### 2.2 Participants

Thirty-two healthy male esports athletes from Germany met the following inclusion criteria: (1) esports athlete defined by being in the top 20% of the in-game ranking system, (2) playing computer-based multiplayer online battle arena (MOBA) or first-person shooter (FPS) games, (3) mouse and keyboard usage, (4) mouse operation with the right hand, (5) using a mouse sensitivity between 400 and 3,000 dots per inch (dpi), (6) aged between 18 and 35 years. The age range reflects the majority of esports athletes (5, 10, 18). Participants were excluded if they reported (1) acute or chronic upper body musculoskeletal disorders, (2) uncorrected visual impairment, (3) severe migraine or epilepsy, (4) medication-induced vigilance or vision impairment, or (5) severe physical or cognitive stress on the previous day. Participants were recruited via social media (*Discord, Instagram, LinkedIn*), in person at video game venues or at various universities in Cologne Germany, as well as through esports organizations. Participant recruitment was open to all genders.

### 2.3 Procedure

The study was conducted by trained and experienced instructors and included subjective and objective parameters. This article will focus on the subjective parameters and procedures. The biomechanical analysis is only partially mentioned to understand the structure of the entire study protocol and will be part of another article. Participants were asked to avoid cognitively or physically demanding activities on the day before and on the day of the test. They were also asked to abstain from alcohol for 12 h, from caffeinated beverages for five hours, and not to use any lotions/creams on the day of the test. At the beginning of the examination, participants were informed about the study protocol and signed the informed consent form. Inclusion and exclusion criteria were then checked, and anthropometric data were recorded. In addition to body weight and height, circumferences, and dimensions of the upper body were collected without clothing. Subsequent recoding of electromyographic, electrocardiographic and motion capture data was prepared. After the preparation for the biomechanical analysis, participants were asked to complete a partially standardized online questionnaire at the testing station.

The standardized test station consisted of an adjustable chair with demounted armrest for a better hip motion capture, an adjustable desk, and ten motion capture cameras*.* While the participants answered the questionnaire, the instructors checked the objective data for plausibility. After completing the questionnaire, participants were allowed to warm up and adjust their settings in the video game for ten minutes. The video game played could be chosen by the participant. The esports title had to be a MOBA (League of Legends, Defense of the Ancients 2) or FPS (Counter-Strike, Valorant, Overwatch, Rainbow Six Siege) video game. Immediately prior to the start of the measurement, participants were asked to do their best to win the games.

After this preparation phase (T0) and at each other measurement point (T1-T3), participants were asked to answer short questionnaires about their current perceived physical state and the current physical exertion. Measurements commenced with the first competitive video gaming session. To ensure typical stress conditions similar to the official competitions, participants had to play ranked games using their main accounts. During the competitive video gaming sessions, participants were asked to rate their perceived physical exertion every 15 min. The sessions ended within 90–120 min, depending on the time each game was finished. Typically, a single game lasted 25–45 min. Therefore, participants had to play multiple games to meet the minimum of 90 min of data collection. If a video game session lasted longer than 120 min, the data recordings for that session were stopped. The competitive video gaming sessions were interrupted by a 10-minute passive sitting break at another chair with armrests. Break duration reflects the average break between tournament games, which may vary between games and tournaments (19–21). Eating and drinking were permitted without restrictions on specific foods or caloric intake. Only caffeinated beverages and smoking were prohibited. Participants were not allowed to be physically active during the break. After the second competitive video game session, a five-minute passive sitting recovery period was part of the study. During this phase, only heart rate monitoring was continued. All other data collection was already completed (Figure 1).

### 2.4 Measuring instruments & outcomes

The questionnaire was designed to assess socio-demographic data, video gaming behavior, physical activity, sitting time, and prevalence of musculoskeletal disorders of esports athletes. It was administered via the online survey tool Unipark (*Questback GmbH, Cologne, Germany*). The questionnaire contained a total of 38–50 questions, depending on participants’ answers to filter questions. First, demographic data such as age, gender, education, and employment status of the participants were collected. The wording and assessment of these questions were designed according to the standards of the German Federal Statistical Office (22). Since an appropriate and validated questionnaire was not available, questions about video game and esports training behaviors were self-designed.

Participants were first asked about their video game genre, their primary video game title and their in-game rank. In order to make the rank distribution of each game comparable, the percentage ranks are given and subdivided: ≤1%, ≤5%, ≤10%, ≤20%. Secondly, the video game experience in years, their mouse dpi and in-game (mouse) sensitivity were queried. Thirdly, they were asked about their video game playing time in hours per week differentiated according by mode:

• “Alone/without human players against human opponents (PvP)”
• “With human players against human opponents (Coop PvP)”
• “Alone/without human players against computer-controlled opponents (PvE)”
• “With human players against computer-controlled opponents (Coop PvE)”

The sum corresponded to the total video game playtime per week. The questionnaire also asked if the participants were a member of an esports club and participated in regular esports training. If they participated in esports training, the follow-up question about the organization of the training contained the following responses:

• “I train in a (regional) club with a coach”
• “I train in a (regional) club without a coach”
• “I train in a team with a coach”
• “I train in a team without a coach”
• “I train with friends”
• “I train alone or with random opponents/teammates”

Multiple answers were possible. In addition, esports training content was asked on a 4-point rating scale (“never”, “sometimes”, “frequently”, “always”):

• Game mechanics
• Tactics
• Game analysis (own games)
• Game analysis (opponents and role models)
• Team building
• Communication with team members
• Reaction speed
• Targeted training of fine motor skills/precision/mechanical skills
• Dealing with stressful situations (in the game)
• Physical fitness
• Relaxation/regeneration
• Other

Participants were additionally queried regarding the proportion of their esports training conducted on PCs and the average weekly training duration in hours. The second part of the questionnaire covered health issues such as overall health, musculoskeletal disorders, physical activity and sitting time. The overall health was observed with a single question and includes the overall health status of the last 4 weeks on a 5-point rating scale: “poor”, “fair”, “good”, “very good”, “excellent”.

Musculoskeletal disorders were evaluated with the validated German version of the *Nordic Musculoskeletal Questionnaire* (NMQ) (23, 24). Physical activity was assessed with the *European Health Interview Survey—Physical Activity Questionnaire* (EHIS-PAQ) (25). The *Sedentary Behavior Questionnaire* (SBQ) was used to assess weekday and weekend seating times (26). The EHIS-PAQ and SBQ were also available in a validated German version.

In addition to this baseline questionnaire, a modified version of the *Borg Categorial-Ratio-10 scale* (CR10) was used to assess only the physical exertion at the measurement points (T0-T3) and every 15-minutes in the competitive video gaming sessions (27). The scale rated the perceived physical exertion from 0 “No physical exertion” to 10 “Extremely strong physical exertion” (Supplementary Material Figure S1). In addition, a German validated list of adjectives was used to assess participants’ current perceived physical state (PEPS) (28). The PEPS is recommended for monitoring changes in perceived physical state during exercise classes to detect short-term changes and was used at measurement points. The assessment is based on a six-point rating scale. Only the endpoints of the scale are verbally anchored (0 = “not at all”; 5 = “completely”). A self-translated English version can be found in the Supplementary Materials.

### 2.5 Sample size

An *a priori* power analysis was performed using G*Power software (version 3.1.9.7) to estimate the sample size required for repeated measures of variance (one-way ANOVA) (29). Due to a lack of scientific evidence, we assumed a mean effect size (f) of 0.25, a significance level (α) of 0.05, and a power (1-β) of 0.8. The analysis included 2 groups (within factors), 4 measurements (T0-T3), a correlation between repeated measures set at 0.5, and a non-sphericity correction (e) of 1. The results indicated a required sample size of *N* = 24.

### 2.6 Statistical methods

All statistical analysis were performed using *R* software (version 4.3.1) (30). Data was checked for completeness, plausibility and outliers. Participants were contacted if plausibility was questionable (e.g., reported >6 h/day of exercise). Outliers were excluded if they were greater or less than three times the standard deviation (31). Descriptive statistics are presented as the mean ± standard deviation (SD).

After this the prerequisites for a repeated measures ANOVA were examined. Normal distribution was visually analyzed at each measurement point for each variable using quantile-quantile (QQ) plots. Normal distribution was assumed if data appears as roughly a straight line. QQ plots for each variable are included in the supplementary (Supplementary Material Figures S2–S7). Sphericity was tested with *Mauchly's test*. If the assumption was violated (*p* ≤ 0.5), the *Greenhouse-Geisser* correction was used. Changes over time were tested by repeated measures ANOVA with *Bonferroni* post-hoc analysis. Effect sizes were calculated by using *Cohen's d* and interpreted as small = 0.2, moderate = 0.5 and large = 0.8 effect (32). The *Friedman* test was used for non-normally distributed data. Multiple pairwise comparisons were estimated using the all-pairs test with exact *p*-values and *Bonferroni* adjustment (33). Effect sizes for Friedmann are calculated only for the overall effect with *Kendall's W*. The coefficient ranges from 0 = indicating no relationship, to 1 = indicating a perfect relationship (34). The significance level for all analyses was set at *p* < 0.05. In line with the open science principle, all data as well as the R-syntax will be available one year after publication and can be found in the supplementary.

## 3 Results

### 3.1 Participants

 Table 1 displays the sample characteristics. In total, 32 male participants, with an average age of 23.8 years (± 3.4), were included in the study without any dropouts. Sociodemographic data revealed that 85% of participants held at least an A-level degree (higher education entrance qualification) and 69% were currently college students. Average physical activity level was 307.8 min/week (± 3.4) and mean sedentary time on workdays was 8.4 h/day (± 3.4). On average, participants spent 3.6 h/day (± 2.0) playing video games, with MOBA being the dominant genre among them with 69%. Every participant achieved a ranking within the top 20% of their respective in-game ranking systems. Additionally, 59% achieved rankings in the top 5% or higher.

**Table 1:** Sample characteristics.

Variables	*N*	Percent	Mean	SD
Anthropometric	32			
Age [years]			23.8	3.4
Height [cm]			180.2	6.7
Weight [kg]			80.8	13.9
Body-mass-index [kg/m^2^]			24.8	3.7
Physical behavior	32			
Physical activity [min/week]			307.8	327.9
Sedentary time workdays [h/day]			8.4	3.4
Sedentary time weekends [h/day]			10.1	3.3
Video game behavior	32			
Video game playtime [h/day]			3.6	1.95
Video game experience [years]			12.6	4.26
Video game genre	32			
MOBA	22	69		
FPS	10	31		
In-game rank distribution	32			
1%	9	28		
5%	10	31		
10%	6	19		
20%	7	22		
Highest degree	32			
Secondary school	1	3		
High school	1	3		
Technical college entry	3	9		
A level	22	69		
University degree	5	16		
Occupation	32			
School student	1	3		
College student	22	69		
Full-time employed	2	6		
Part-time employed	4	12		
Marginal employed	1	3		
Vocational training	1	3		
Unemployed	1	3		

The musculoskeletal complaints with all temporal prevalences can be found in Table 2. With regard to the one-year prevalence of musculoskeletal complaints, neck discomfort was the most common complaint among the participants (Table 2). Hand and wrist discomfort were the most common complaints for both four-week and seven-day prevalence.

**Table 2:** Prevalences of musculoskeletal disorders for different body parts.

Body part	One-year prevalence *n* (%)	Restricted by pain last year *n* (%)	Four-week prevalence *n* (%)	Seven-day prevalence *n* (%)
Neck	16 (50.0)	2 (6.3)	5 (15.6)	2 (6.3)
Shoulders and upper arms	7 (21.9)	3 (9.4)	3 (9.4)	2 (6.3)
Elbows and forearms	4 (12.5)	2 (6.3)	2 (6.3)	0 (0.0)
Hands and wrists	9 (28.1)	3 (9.4)	6 (18.8)	4 (12.5)
Thoracic spine	10 (31.3)	0 (0.0)	3 (9.4)	1 (3.1)
Lumbar spine	10 (31.3)	5 (15.6)	4 (12.5)	1 (3.1)
Hip joints and thighs	3 (9.4)	2 (6.3)	2 (6.3)	1 (3.1)
Knee joints	4 (12.5)	1 (3.1)	3 (9.4)	3 (9.4)
Lower leg	4 (12.5)	2 (6.3)	1 (3.1)	0 (0.0)
Feet and ankles	5 (15.6)	3 (9.4)	4 (12.5)	2 (6.3)

**Figure 2:**
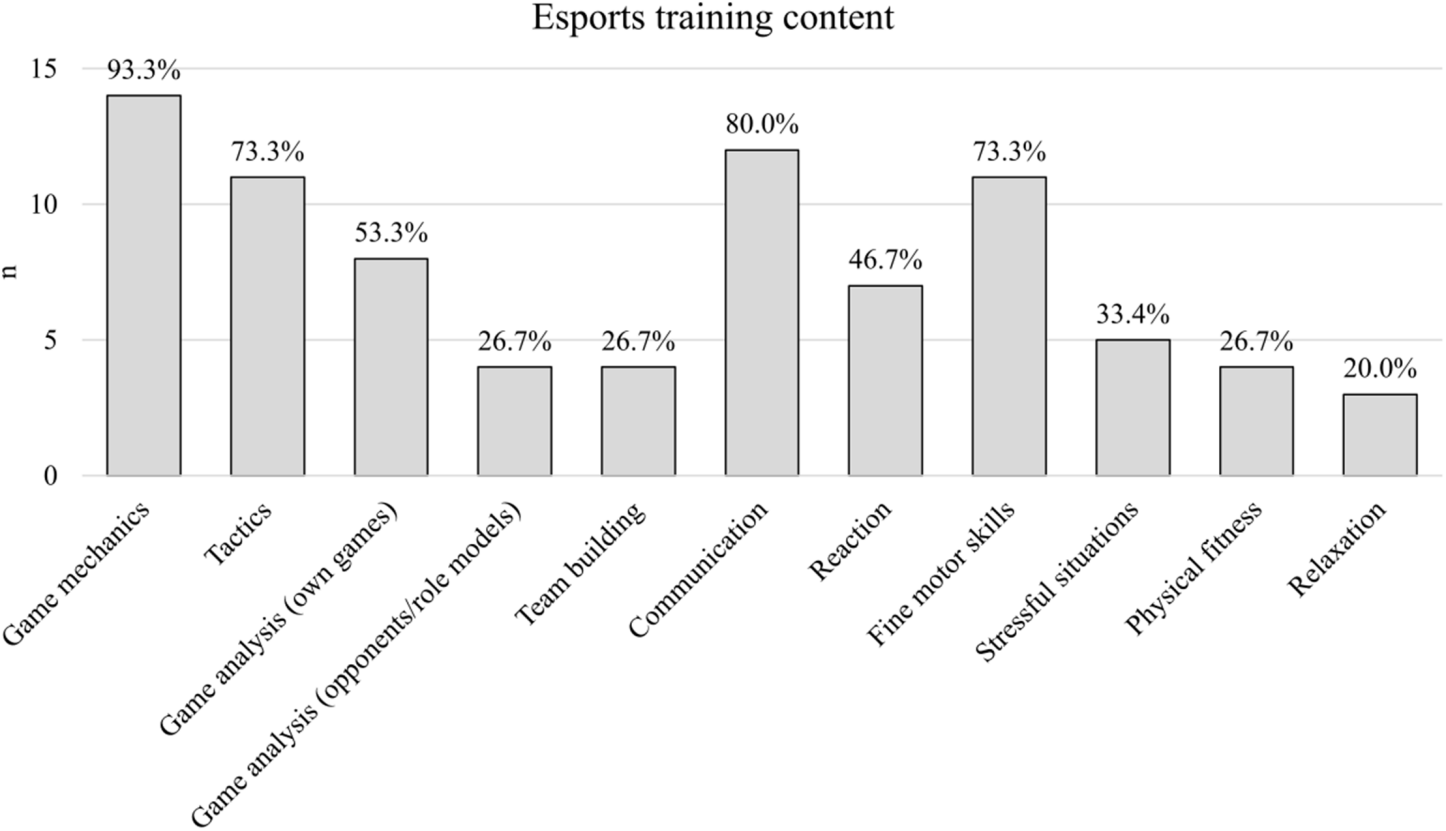
Content of esports athletes’ structured trainings (*n* = 15). Values represented participants who stated that they frequently or always train for that aspect in their training sessions.

Figure 2 shows the exact training content. Only 15 out of 32 esports athletes participate in regular esports training. They are most likely to train either alone (53.3%), in a team (53.3%), in a team with a coach (40.0%) or with friends (40.0%). There is minimal training with an esports club (26.7%) or with a club and with a coach (6.7%).

### 3.2 Perceived physical state

**Figure 3:**
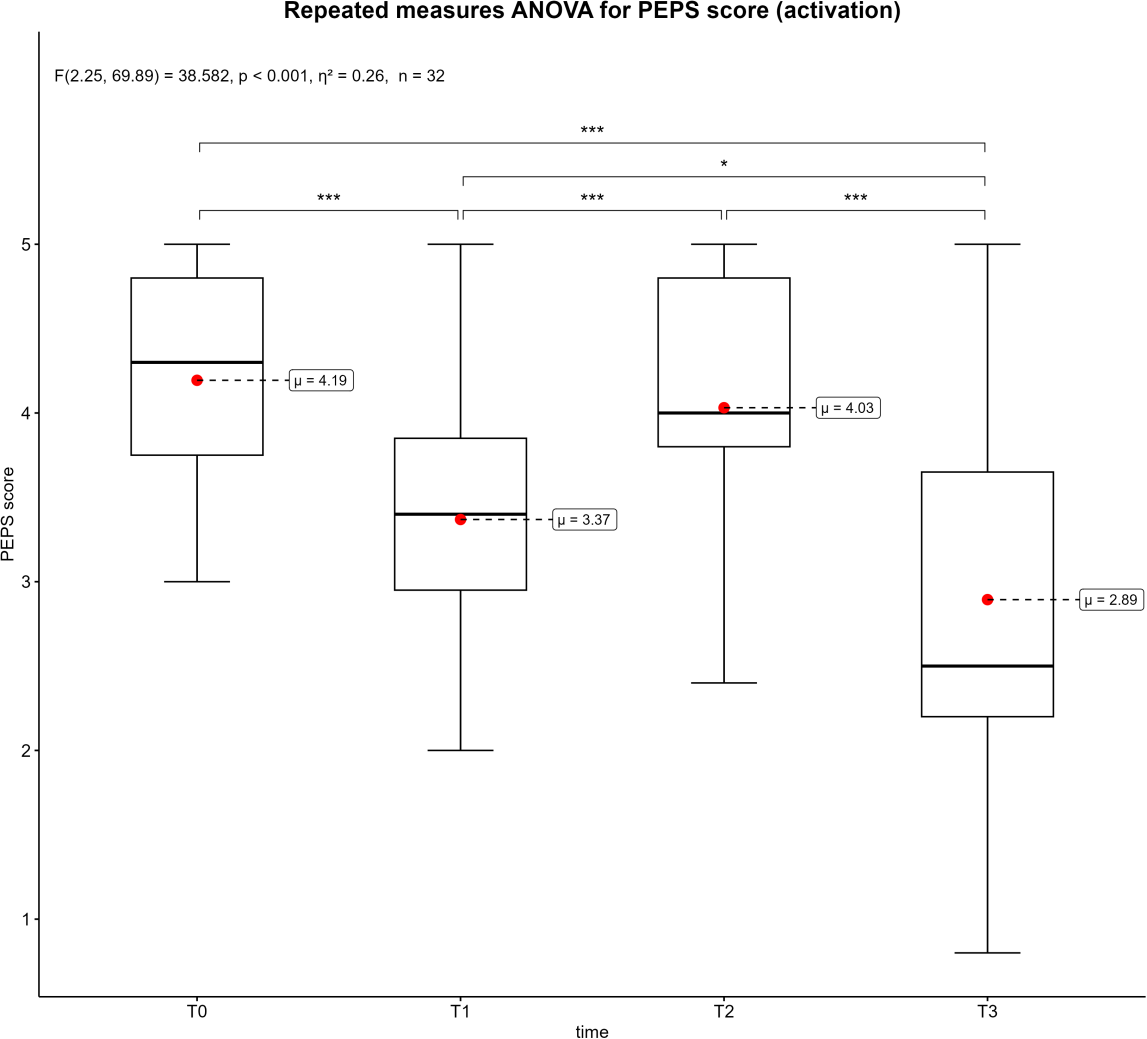
Box plots of the PEPS dimension activation across the measurement points. T0 = baseline, T1 = after the first competitive video gaming session, T2 = after the ten-minute break, T3 = after the second competitive video gaming session. *: *p* ≤ 0.05, **: *p* ≤ 0.01, ***: *p* ≤ 0.001 with *Greenhouse-Geisser* correction.

**Figure 4:**
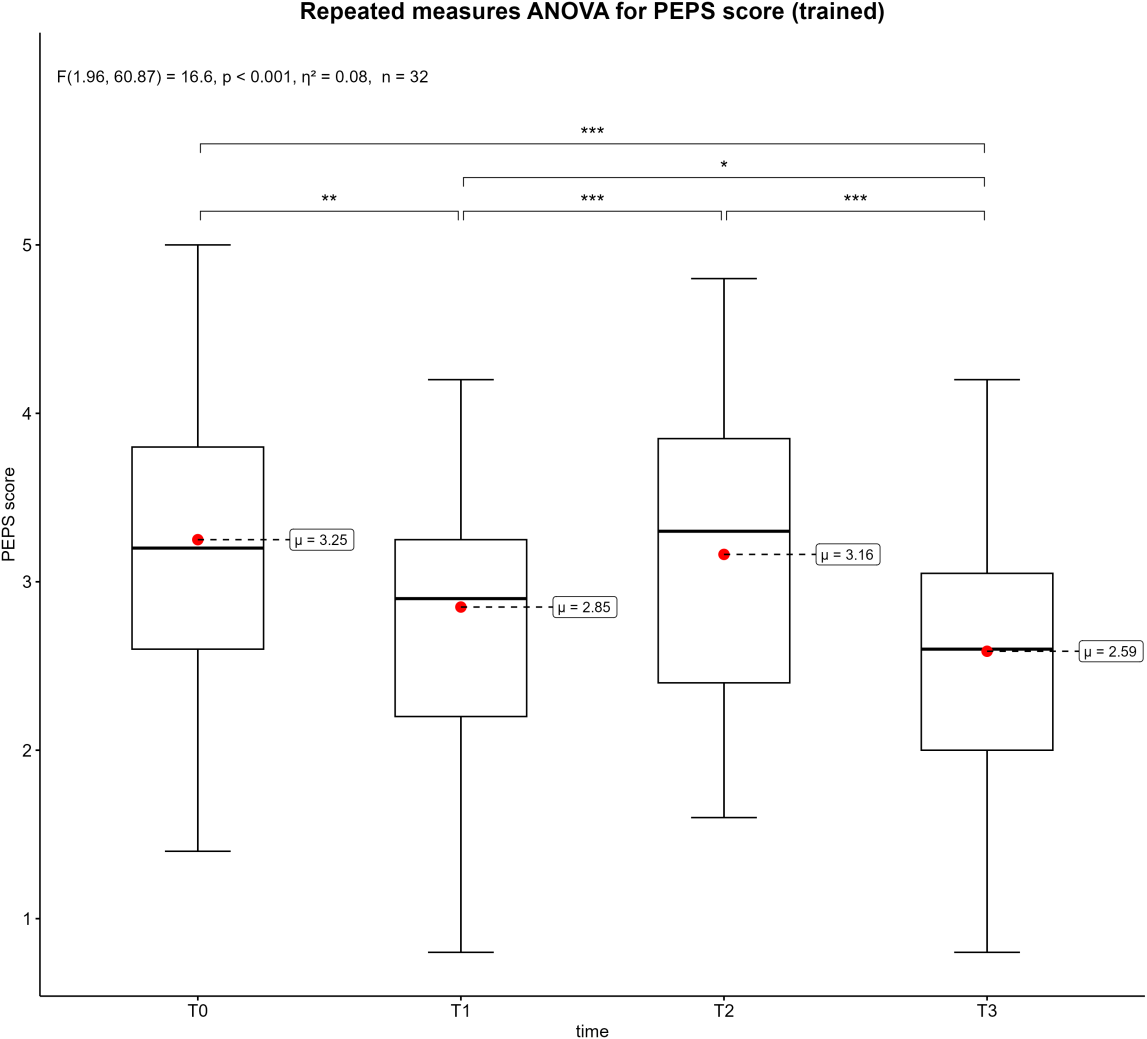
Box plots of the PEPS dimension trained across the measurement points. T0 = baseline, T1 = after the first competitive video gaming session, T2 = after the ten-minute break, T3 = after the second competitive video gaming session. *: *p* ≤ 0.05, **: *p* ≤ 0.01, ***: *p* ≤ 0.001, with *Greenhouse-Geisser* correction.

**Figure 5:**
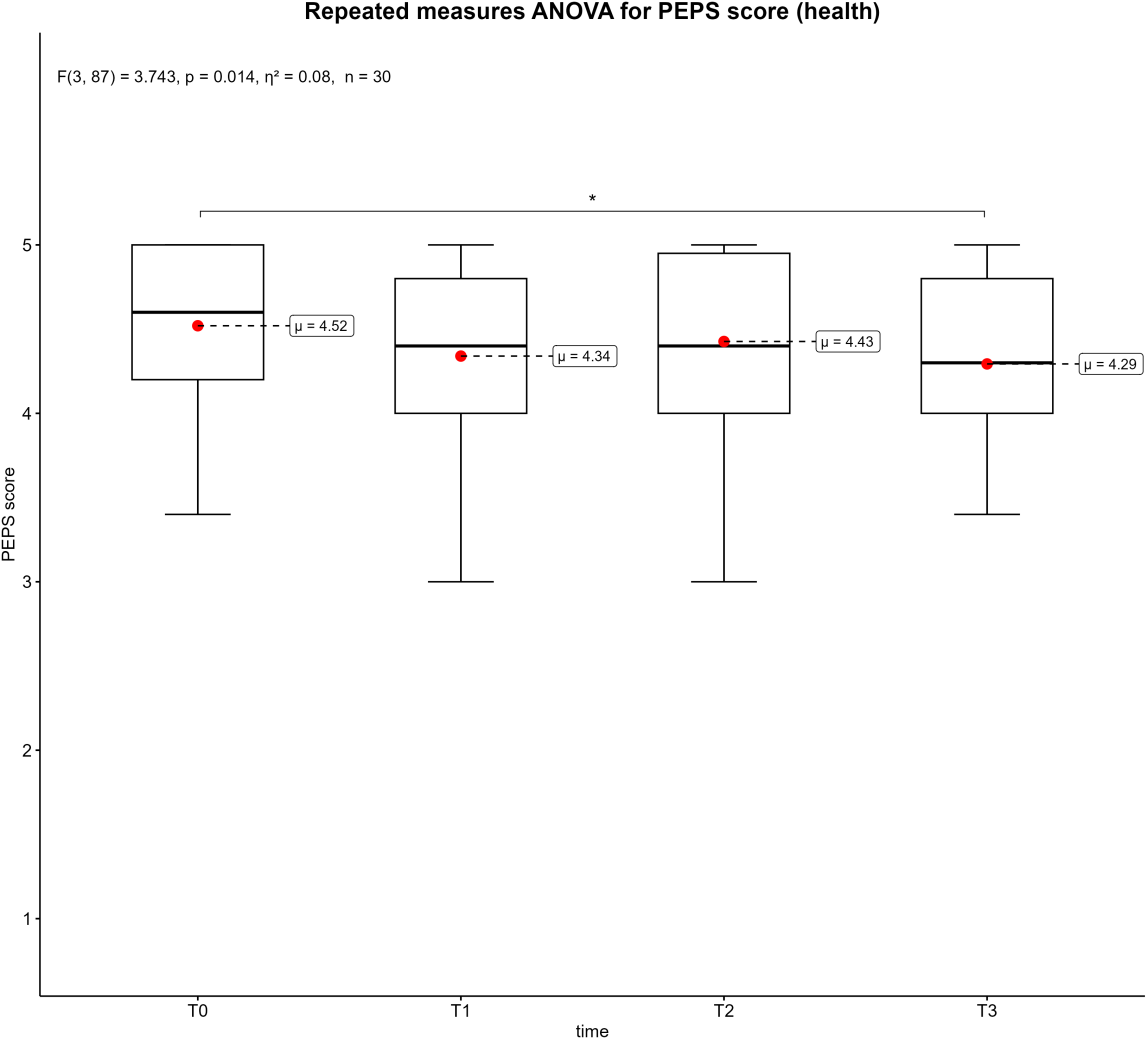
Box plots of the PEPS dimension health across the measurement points. T0 = baseline, T1 = after the first competitive video gaming session, T2 = after the ten-minute break, T3 = after the second competitive video gaming session. *: *p* ≤ 0.05, **: *p* ≤ 0.01, ***: *p* ≤ 0.001.

**Figure 6:**
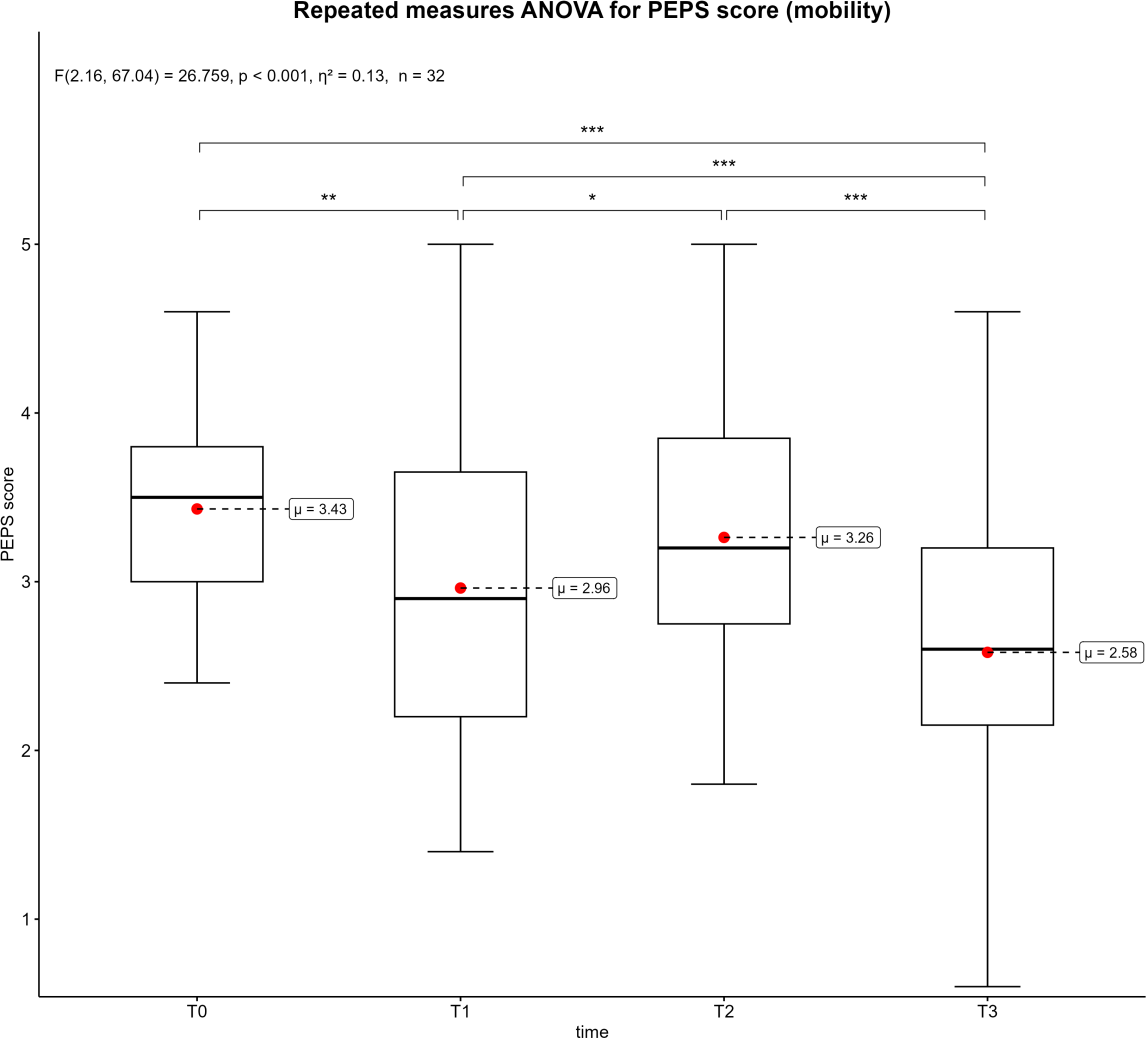
Box plots of the PEPS dimension mobility across the measurement points. T0 = baseline, T1 = after the first competitive video gaming session, T2 = after the ten-minute break, T3 = after the second competitive video gaming session. *: *p* ≤ 0.05, **: *p* ≤ 0.01, ***: *p* ≤ 0.001, with *Greenhouse-Geisser* correction.

Figures 3–6 displays the box plots of the perceived physical condition during the competitive video gaming sessions. The results of the ANOVA with repeated measures show a significant difference in all dimensions of the PEPS: activation (*p* < 0.001, *η*^2^ = 0.26), trained (*p* < 0.001, *η*^2^ = 0.08), health (*p* = 0.014, *η*^2^ = 0.08) and mobility (*p* < 0.001, *η*^2^ = 0.13). However, the post-hoc tests revealed that only T0 differs from T3 in the health dimension (*p* = 0.039). In the other three dimensions, all measurement times differ significantly from each other with exception of T0 to T2. Overall, there was a decrease over time. The activation dimension went from 4.19 ± 0.62 at T0 to 2.89 ± 1.16 at T3 (−26%). The trained dimension decreased from 3.25 ± 0.85 to 2.59 ± 0.85 (−13.2%) and mobility dimension from 3.43 ± 0.67 to 2.58 ± 0.93 (−17%). In addition, all show a moderate to large effect size. The results of all post-hoc tests are shown in in the supplementary (Supplementary Material Table S1).

### 3.3 Borg scale

**Figure 7:**
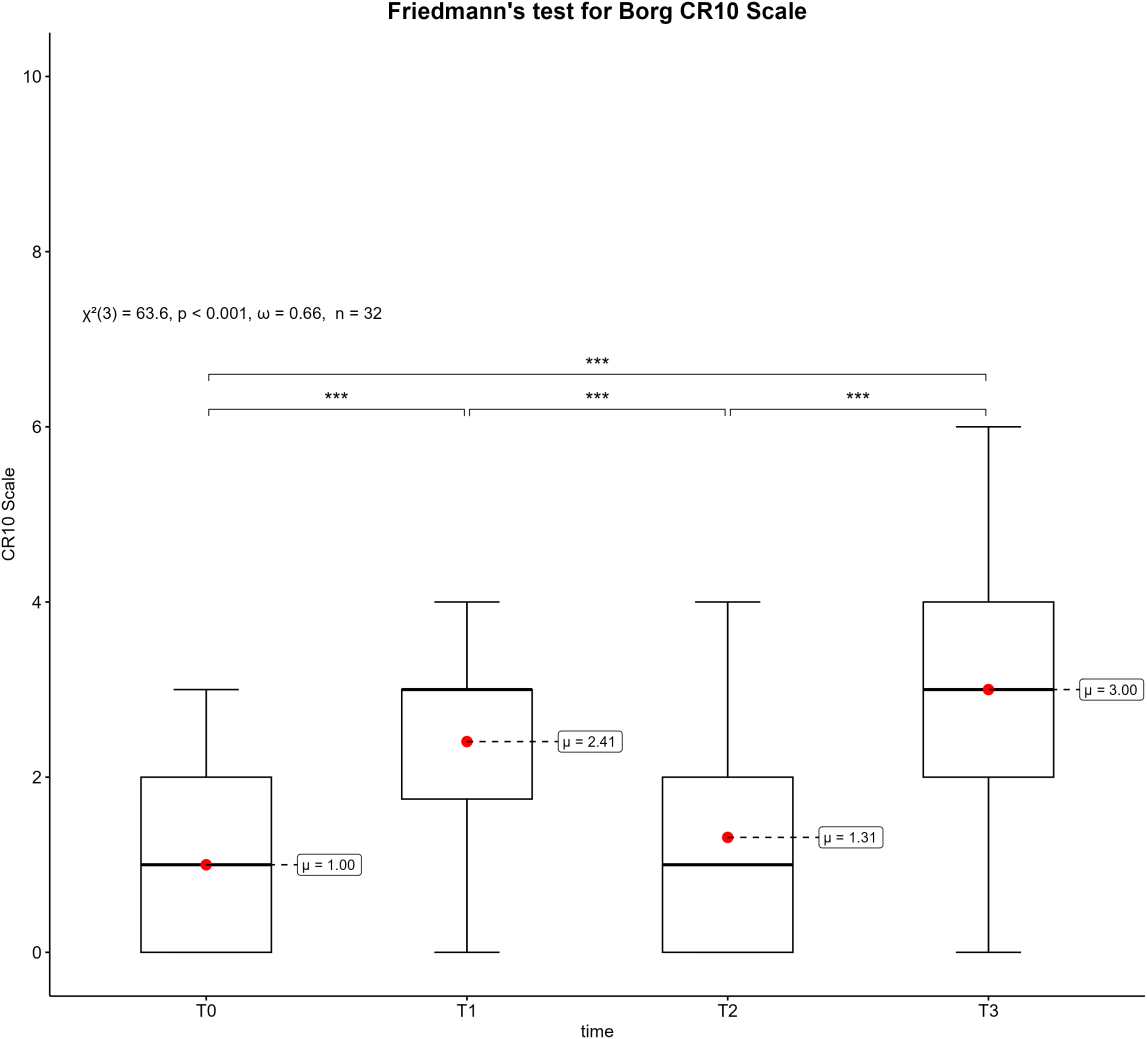
Box plots of the borg scale across the measurement points. T0 = baseline, T1 = after the first competitive video gaming session, T2 = after the ten-minute break, T3 = after the second competitive video gaming session. *: *p* ≤ 0.05, **: *p* ≤ 0.01, ***: *p* ≤ 0.001.

Figure 7 shows the boxplots of the Borg scale at the four measurement points. The Friedmann test indicates significant differences between the measurement times according to the Borg scale (*p* < 0.001, *ω* = 0.66). The post-hoc tests revealed significant differences between baseline (T0) measurements (1.0 ± 1.0) and after the first (T1) competitive video gaming session (2.4 ± 1.3, *p *< 0.001) as well as after the second (T3) competitive video gaming session (3 ± 1.7, *p* < 0.001). Accordingly, Borg scale increased by 2 points over the entire measurement, which corresponds to an increase of 20%. Furthermore, there was a significant difference between the measurement time after the first competitive video gaming session and the break (T2) (1.3 ± 1.2, *p* < 0.001). Lastly, there was also a significant difference between T2 and T3 (*p *< 0.001).

**Figure 8:**
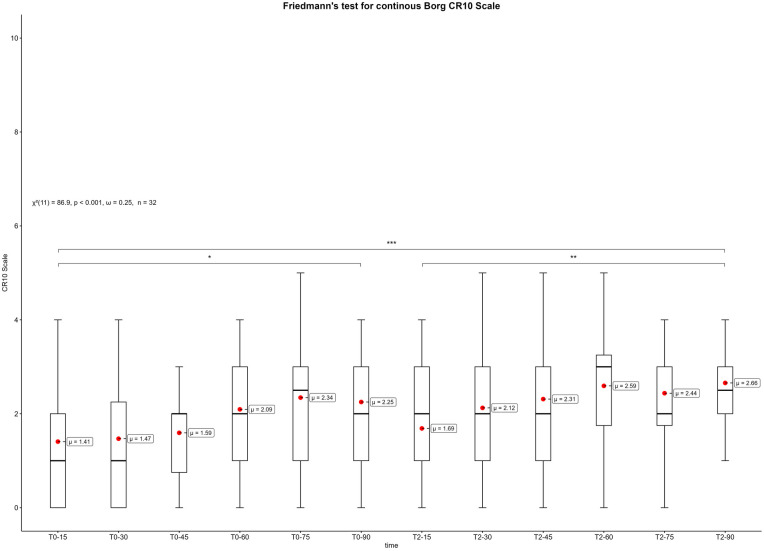
Box plots of the borg scale during the competitive video gaming sessions every 15 min. T0-min = first competitive video gaming session, T2-min = second competitive video gaming session. *: *p* ≤ 0.05, **: *p* ≤ 0.01, ***: *p* ≤ 0.001.

Considering the measurement times of the borg scale every 15 min during the competitive video gaming sessions, the results of the Friedmann test also show significant differences (*p *< 0.001, *ω* = 0.25). Figure 8 displays the box plots of the borg scale with measurement points every 15 min during the competitive video gaming sessions. For reasons of clarity, only the most important significances are shown in the figure. The results of the post-hoc tests between all time points can be found in the supplementary (Supplementary Material Table S3).

## 4 Discussion

The purpose of this study was to examine the perceived physical burdens of esports athletes. Thirty-two male esports athletes participated in two 90–120-minute competitive video gaming sessions and reported their perceived physical exertion and perceived physical state. The main finding of this study is that the perceived physical burdens significantly increase during esports. As the duration of competitive video gaming extends, the perceived physical state decreased and the perceived physical exertion increased. Therefore, the hypothesis can be confirmed. However, a 10-minute passive break between competitive video gaming sessions only temporarily reduced perceived physical burdens.

### 4.1 Perceived physical burdens in esports

Each PEPS dimension was associated with significant decreases over time (see Figures 3–6). The largest decrease was recorded in the activation dimension (−26%) and the lowest in health (−4%). With exception of health, every dimension also indicated significant changes between measurement points. It seems logical that a complex and solid construct like health would not be affected by a temporary mental and sedentary activity like esports. In addition, the control variables “physical pain” and “physical discomfort”, which are related to the health dimension (28), did not show a significant change between measurement points (Supplementary Material Table S2). A possible reason could be the short duration of video gaming (3–4 h), which might not be sufficient to develop pain or health issues. Additionally, musculoskeletal disorders are often a result of chronicity, which takes time to develop (35, 36). Therefore, playing video games repeatedly for extended periods could potentially impact physical health and the perceived physical state (14). The other PEPS dimensions exhibited similar changes of the PEPS scale. A decrease after both competitive video gaming sessions and a recovery after the break, with the decrease in the second phase being greater than in the first. This could indicate that a 10-minute break between two competitive video gaming sessions could have a positive impact on perceived physical state. However, this break does not restore the PEPS scores to their baseline levels, nor does it prevent a further decline in PEPS scores during the subsequent video game session. In particular, the second session (T2-T3) showed large effect sizes in all PEPS dimensions except the health dimension (Supplementary Material Table S1). Consequently, regular breaks could have a beneficial effect on perceived physical burdens but cannot prevent esports athletes from an increase of these perceived burdens over time. The duration or type of breaks as well as the accumulation of loads could explain this. In relation to different types of breaks, similar results were shown for executive function (37). This study compared walking, sitting, supine rest and no break between 60 and 75 min of FPS gaming. The results suggest that walking and continuous play lead to significantly better executive function scores than supine rest (37). The results of a recently published review, which summarized the positive effects of active breaks in sedentary adults, are partially consistent with these findings (38). According to the authors, metabolic, cardiovascular, and cognitive improvements are associated with light to moderate physical activity or intermittent standing. At the same time, active breaks may mitigate abnormal vascular and hormonal changes which are associated with excessive sitting (38). Consequently, regular breaks could not only improve performance of esports athletes, but also benefit their health and body perception. Specifically, the implementation of active break routines should be strongly encouraged.

The distribution pattern of Borg ratings at measurement points is similar to that of the PEPS ratings. The reverse scaling should be taken into account. Therefore, the perceived physical exertion increased significantly during competitive video gaming sessions and decreased after the break (Figure 7). The overall (T0-T3) increase in mean Borg scale was from “very weak” (=1) to “moderate” (=3). More detailed insights were gathered from continuous Borg scores during competitive video gaming (Figure 8). The values fluctuate and do not form a linear increase. Unexpectedly, the highest Borg score of the first session was reached at the penultimate measurement point (T0-75). Similarly, a higher score was achieved in the second phase at T2-60 than at T2-75. The nature of competitive video gaming may be the reason. In order to compete with other esports athletes of the same skill level, competitors must join queues. Depending on their skill level and the availability of other esports athletes, the queue time can vary (39). This can result in higher scattering and different peaks of Borg scale. But even 90-minutes of competitive video gaming significantly increased the Borg scores. Thus, 3–4 h of esports noticeable increase the perceived physical exertion. In addition, a 10-minute break can provide short-term recovery from physical exertion. However, compared to esports training durations of up to 11 h/day (40) or tournament conditions it is concerning that even this shorter duration of competitive video gaming produces such significant changes. As mentioned above, loads could accumulate and lead to higher perceived exertions and burdens over time. Only one other study used Borg scale with video gamers, but only after playing (37). The study showed Borg scores on the original scale (6–20) with a mean of 11.3–13.4, indicating “fairly light” to “somewhat hard” intensities. In this case, the highest scores were reached after continuous, uninterrupted video game play, but without significant differences from the other groups (37). Thus, the ratings are similar to the Borg scale, but they differ in terms of methodology. What distinguishes the present study is the application of time series analysis to the Borg scale. However, this is an indication of the perceived burdens that playing video games places on esports athletes. Related results were found for prolonged sitting for 4 h and an increase in perceived discomfort in different body parts (41). This could be a possible reason for an increase in the Borg score, but as mentioned above, physical discomfort or pain did not increase significantly in the present study. Therefore, it can be assumed that the Borg score increased independently of discomfort or pain. In conclusion, in the current study esports athletes perceived moderate physical exertion after 3–4 h of competitive video gaming. In addition, this study shows that a passive break between two sessions can at least partially restore physical exertion and physical state. Nevertheless, future research should evaluate various types of breaks and break durations to gain a better understanding of their potential health and performance benefits. This understanding can then be used to implement breaks into esports training in a more meaningful manner.

### 4.2 Limitations and strengths

The results of this work should be understood in the context of certain limitations. The study was designed without a control group or comparison, which limits the causality and may lead to biased results. In addition, competitive video game time ranged between 90 and 120 min per session. Therefore, some participants played longer periods of time, which can affect the results. In contrast, during these competitive video game sessions, participants had to wait in queue for their games. This queue time was not recorded but can vary from few seconds up to 10 min. This time often depends on the rank of the esports athletes and increases with rank. As result, some participants had less time to play competitively. Moreover, esports athletes out of different video game genres (MOBA, FPS) were included, due to the suspected similar exposure. Because of the sample size, the groups were not compared and the statistical models were not adjusted for this. Furthermore, no validated measuring instrument for perceived physical burdens in esports exists. Therefore, measuring instrument were used that are validated, but originally designed for physically active behavior. This can result in bias. In addition, the interpretation of the PEPS dimension activation should also be viewed critically. This dimension consists of the adjectives energy less, exhausted, drained, flabby, and limp, and could also be associated with mental processes. Mental capacity could easily be affected by mental workload, such as esports. This could lead to less differentiation between mental and physical activation after competitive video gaming sessions. In contrast, (light) physical activity results in increased scores on the activation dimension (28), which could be due to physical or psychological factors. Additionally, only male esports athletes registered for this study. Therefore, the recruitment strategy should have been modified to attempt to improve the recruitment rate of non-male esports athletes and to avoid gender bias.

However, this study showed for the first time how esports athletes perceive physical burdens under realistic conditions in a controlled setup. This will contribute to the understanding of internal and external workloads associated with esports competition and training. In addition, the data sample size is strong for interventional esports research.

### 4.3 Practical implications

It is important to consider these results when structuring training programs for esports athletes. Regular breaks should be included in any esports training routine to avoid an increase in perceived physical burdens. In this study, passive breaks at least partially restored physical exertion and physical state. To enhance this effect and improve health and performance, physical activity should be a part of these breaks (42, 43). Even a 6-minute walk can improve cognitive function and subjective well-being in esports athletes (37).

Furthermore, body perception and perception of exhaustion should be trained. This could potentially empower esports athletes and coaches in load management and monitoring. In particular, coaches and health professionals should implement regular monitoring of these conditions in order to adjust training and health programs. As result, performance declines and health issues could be prevented or counteracted at an early stage. Additional objective measures, such as heart rate variability, eye tracking, or electromyography, could be beneficial as comparative parameters.

## 5 Conclusion

In summary, competitive video gaming of 3–4 h can negatively affect the perceived physical exertion and the perceived physical state of esports athletes. A passive break may provide short-term regeneration but cannot fully restore. Over time and with a lack of observation, this could result in health and performance limitations. In addition, breaks should incorporate physical activity to mitigate the additional negative consequences of sedentary behavior, such as in esports. Moreover, physical exercise and body perception should be a crucial part of esports training. For practical implications, esports athletes are recommended to regularly monitor their burden and exertion, especially during competitive video gaming. This could lead to improve body perception, which is essential in preventing overtraining, overuse injuries, and burnout. Therefore, further research should focus on examining the validity and reliability of common measures of (perceived) exertion in esports. Additionally, more studies are needed to objectively investigate the physical burdens experienced during competitive video gaming.

## Data Availability

The datasets presented in this study can be found in online repositories. The names of the repository/repositories and accession number(s) can be found in the article/**Supplementary Material**.
