# Supplementary material for: How strenuous is esports? Perceived physical exertion and physical state during competitive video gaming
Source: Front Sports Act Living. 2024 Jul 10;6:1370485. doi: 10.3389/fspor.2024.1370485 (PMC11266069; doi:10.3389/fspor.2024.1370485)
Supplement: Supplementary file 1 [file Datasheet1.pdf]

Supplementary Material

How strenuous is esports? Perceived physical exertion and physical state during competitive video gaming

Chuck Tholl*, **Markus Soffner, Ingo Froböse**

*** Correspondence:** Corresponding Author: c.tholl@dshs-koeln.de

1 Supplementary Data

The raw data supporting the conclusions of this article will be made available by the authors one year after publication. All data and materials from this study are available on the *Open Science Framework* (DOI: 10.17605/OSF.IO/9QB6H, https://osf.io/9qb6h/).

2 Supplementary Figures and Tables

2.1 Supplementary Figures

0	No physical exertion
1	Very weak
2	Weak
3	Moderate
4	
5	Strong
6	
7	Very strong
8	
9	
10	Extremely strong physical exertion

Supplementary Figure 1: Modified Borg Categorial-Ratio-10 scale.

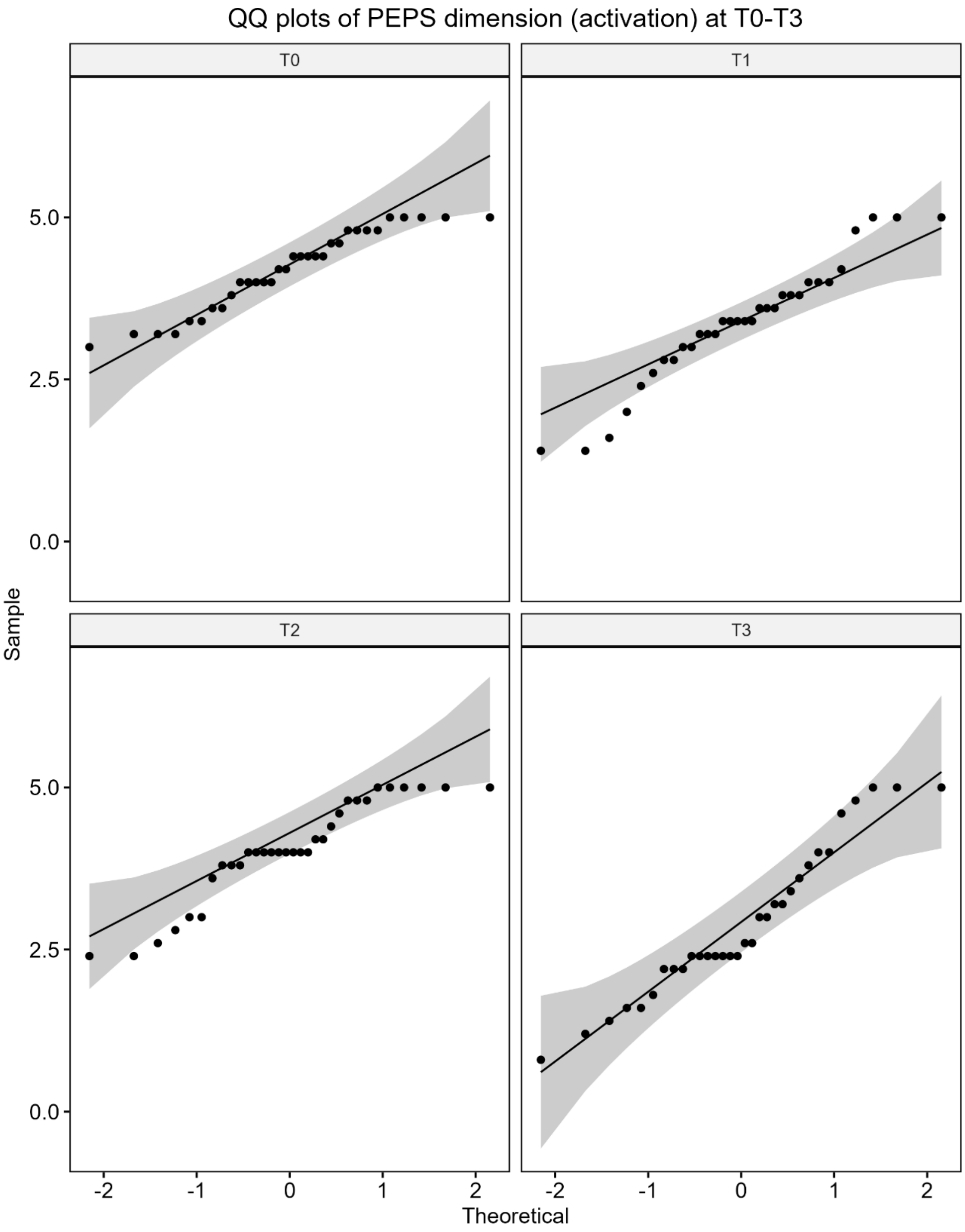

Supplementary Figure 2: Normal distribution of PEPS dimension (activation) at measurement points (T0-T3). T0 = baseline, T1 = after the first competitive video gaming session, T2 = after the ten-minute break, T3 = after the second competitive video gaming session.

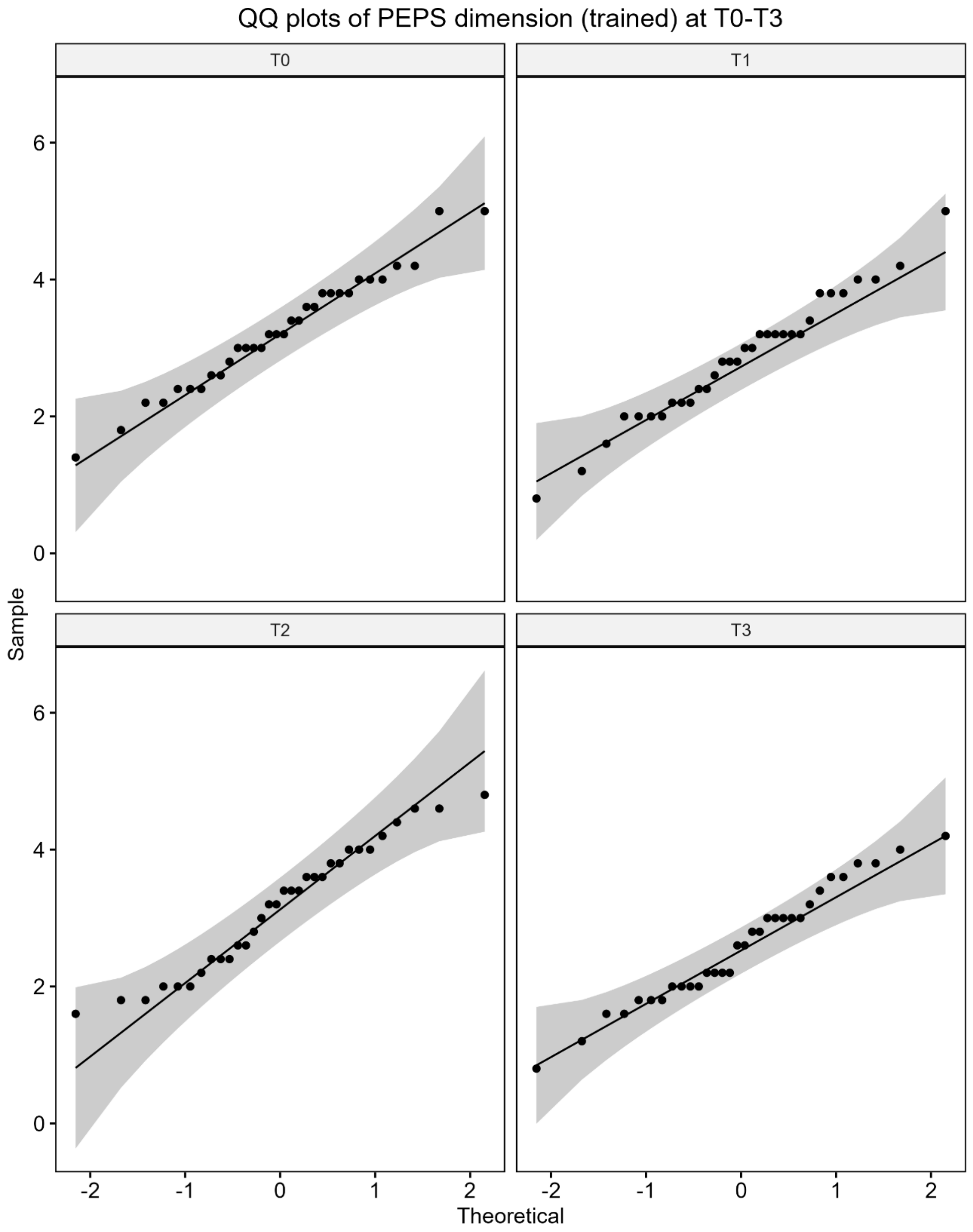

Supplementary Figure 3: Normal distribution of PEPS dimension (trained) at measurement points (T0-T3). T0 = baseline, T1 = after the first competitive video gaming session, T2 = after the ten-minute break, T3 = after the second competitive video gaming session.

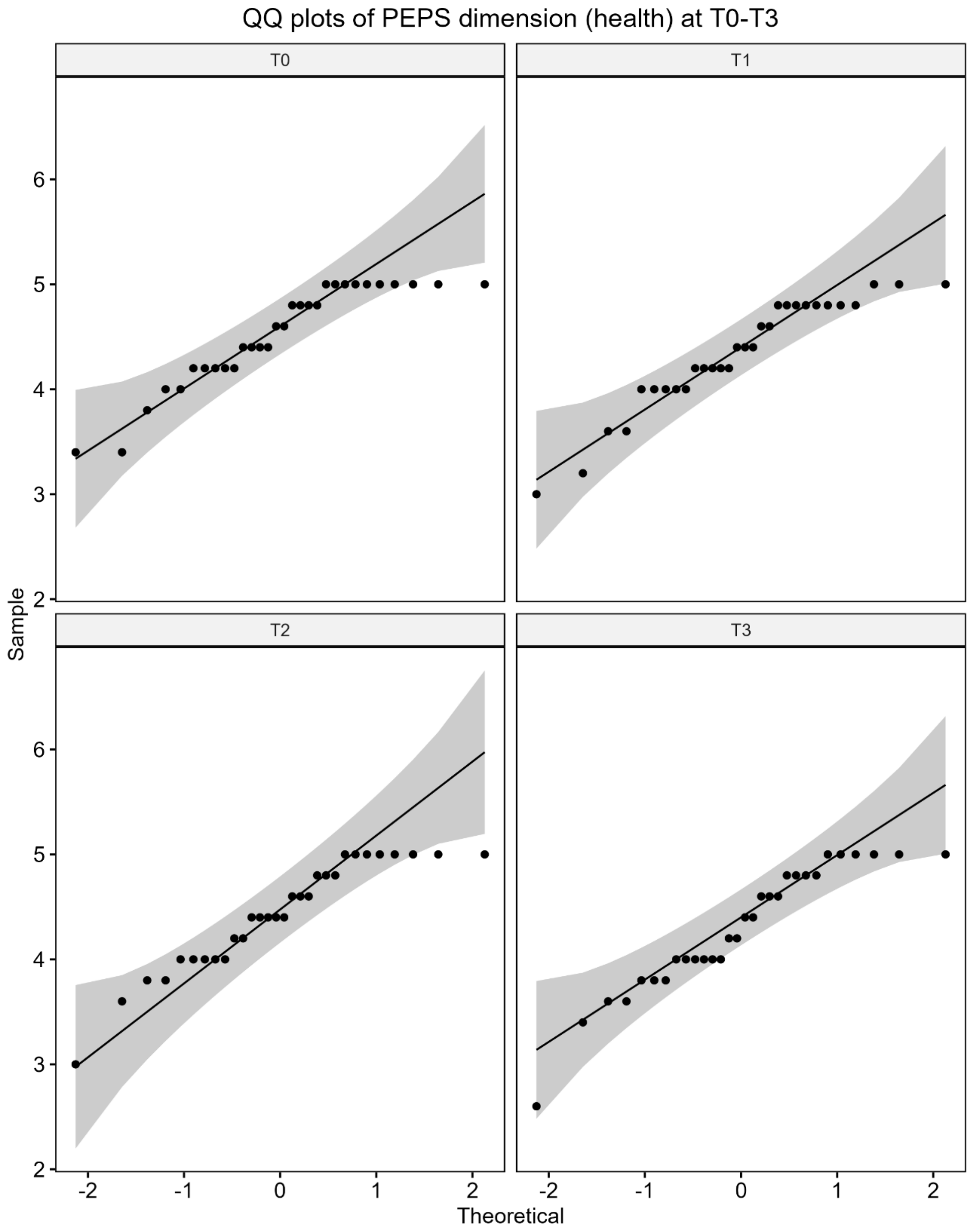

Supplementary Figure 4: Normal distribution of PEPS dimension (health) at measurement points (T0-T3). T0 = baseline, T1 = after the first competitive video gaming session, T2 = after the ten-minute break, T3 = after the second competitive video gaming session.

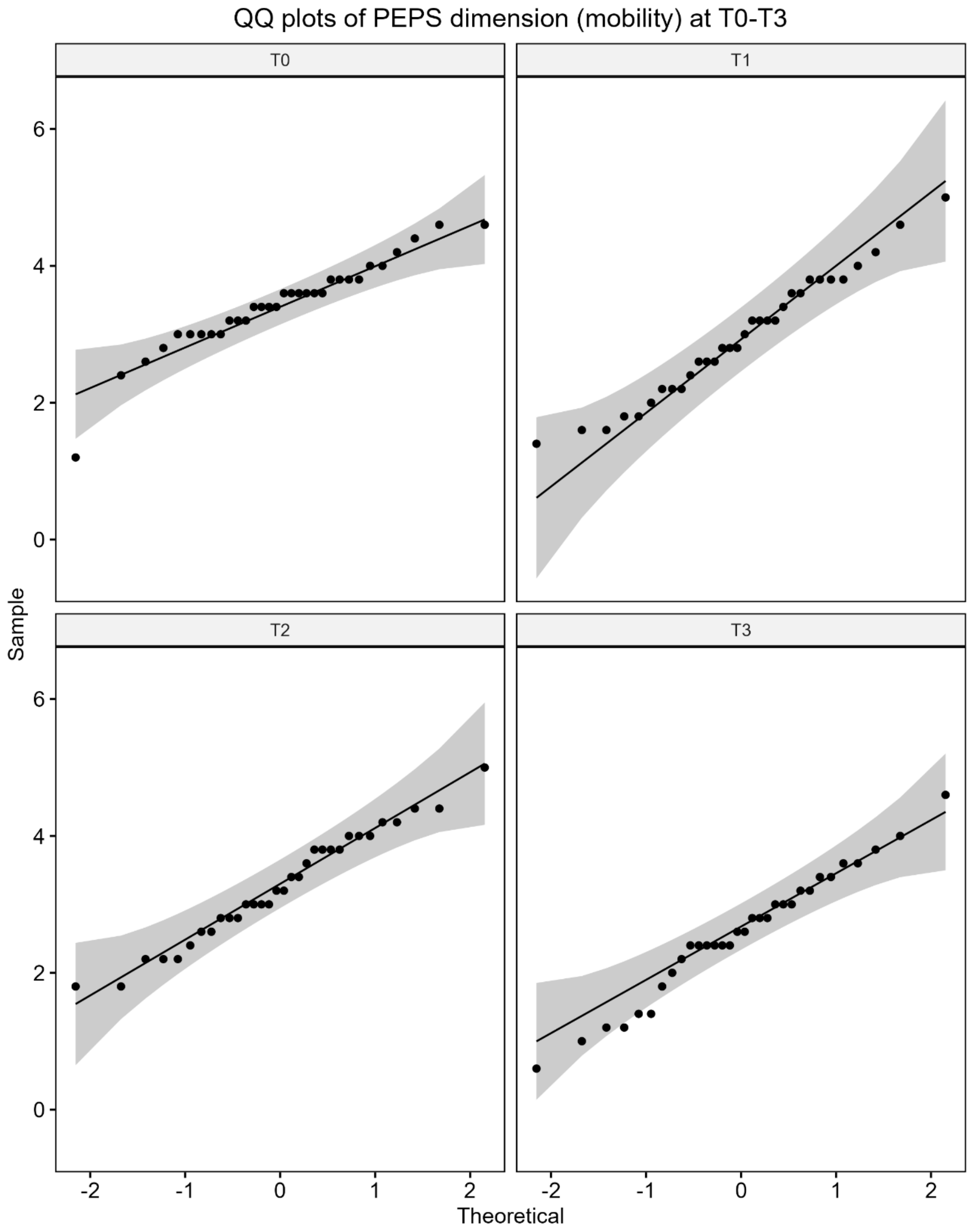

Supplementary Figure 5: Normal distribution of PEPS dimension (mobility) at measurement points (T0-T3). T0 = baseline, T1 = after the first competitive video gaming session, T2 = after the ten-minute break, T3 = after the second competitive video gaming session.

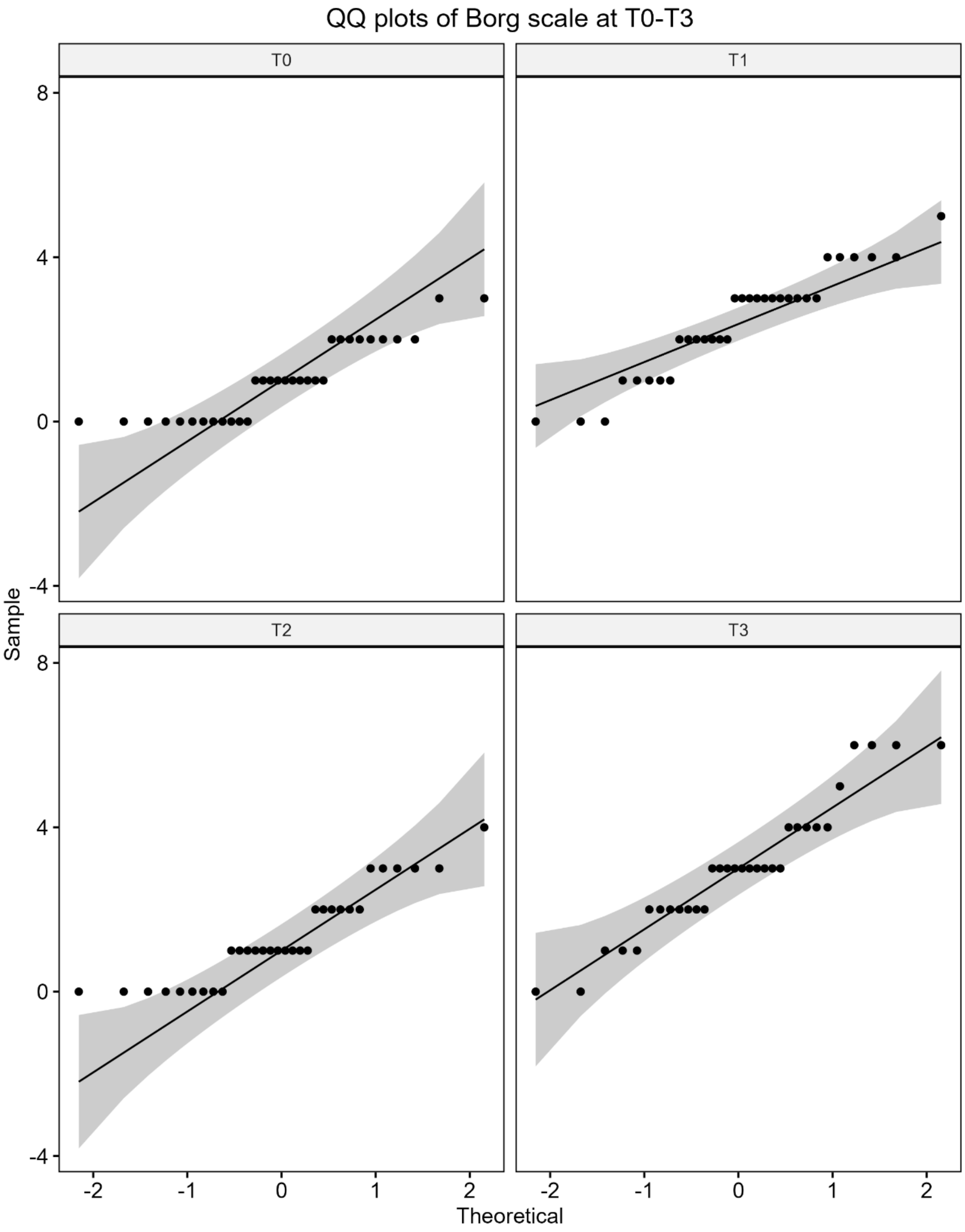

Supplementary Figure 6: Normal distribution of Borg scale at measurement points (T0-T3). T0 = baseline, T1 = after the first competitive video gaming session, T2 = after the ten-minute break, T3 = after the second competitive video gaming session.

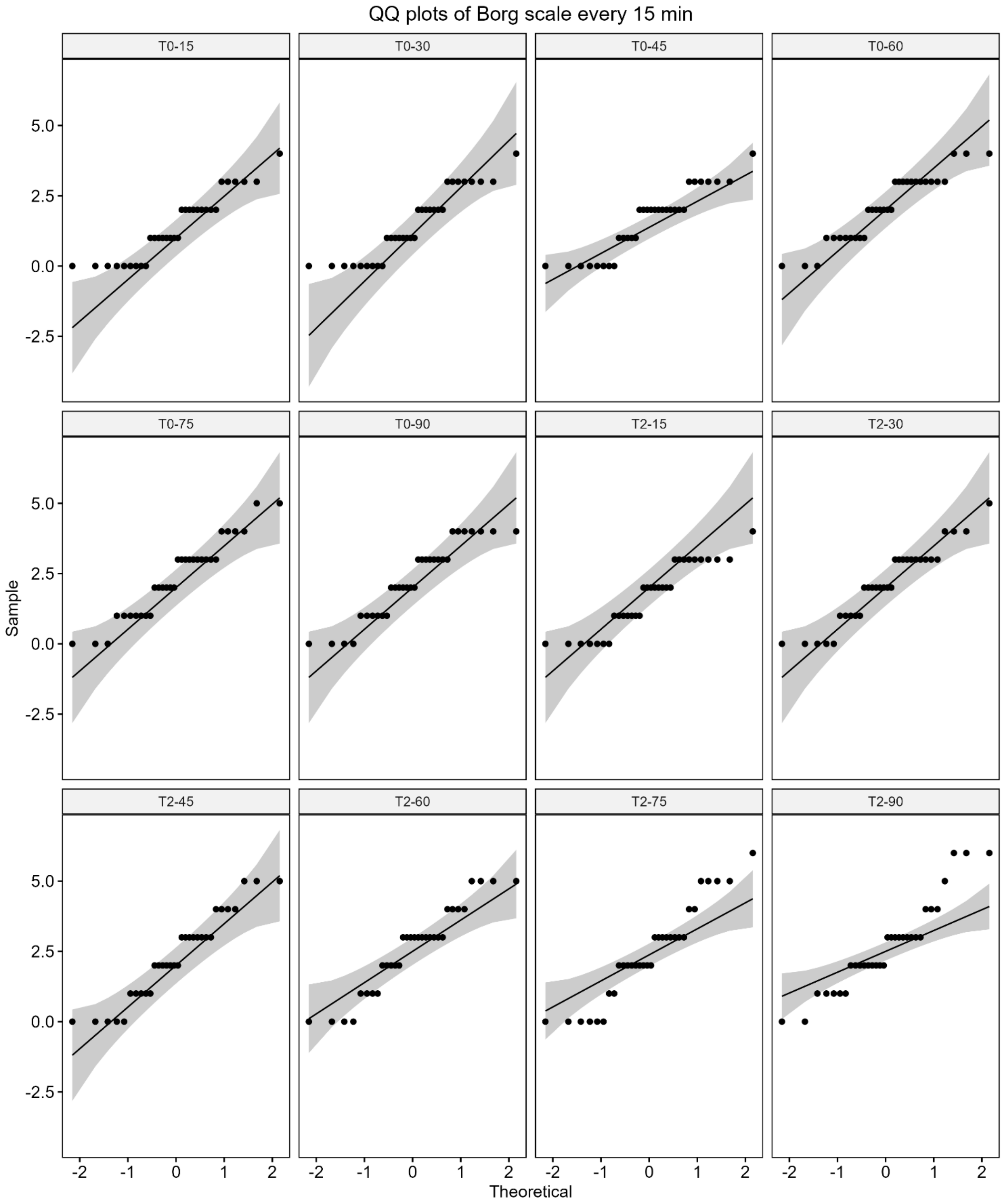

Supplementary Figure 7: Normal distribution of Borg scale every 15 minutes during competitive gaming. T0-min = first competitive video gaming session, T2-min = second competitive video gaming session.

2.2 Supplementary Tables

Supplementary Table 1. Repeated measures ANOVA and effect sizes of PEPS dimensions.

	**Activation** F	**Trained** F	**Health** F	**Mobility** F
**T0 – T1**	7.18	3.71	2.21	3.93
p (η^2)	< 0.001 (1.27)	< 0.001 (0.66)	0.21 (0.40)	< 0.01 (0.70)
**T0 – T2**	1.70	0.95	1.17	2.01
p (η^2)	0.60 (0.30)	0.35 (0.17)	1.00 (0.21)	0.32 (0.36)
**T0 – T3**	7.51	4.49	2.93	6.96
p (η^2)	< 0.001 (1.33)	< 0.001 (0.79)	0.039 (0.54)	< 0.001 (1.23)
**T1 – T2**	-5.44	-4.32	-1.51	-3.36
p (η^2)	< 0.001 (-0.96)	< 0.01 (-0.76)	0.85 (-0.28)	0.01 (-0.59)
**T1 – T3**	3.23	2.96	0.72	4.96
p (η^2)	0.02 (0.57)	< 0.01 (0.52)	1.00 (0.13)	< 0.001 (0.88)
**T2 – T3**	7.30	5.34	1.78	6.12
p (η^2)	< 0.001 (1.28)	< 0.001 (0.94)	0.52 (0.32)	< 0.001 (1.08)

T0 = baseline, T1 = after the first competitive video gaming session, T2 = after the ten-minute break, T3 = after the second competitive video gaming session.

Supplementary Table 2. Friedmann test for PEPS control variables with post-hoc analysis (mean ± standard deviation).

	Discomfort		Pain	
**T0 – T1**	0,31 ± 0,69<br>0,75 ± 0,95	p = 0.17	0,13 ± 0,55<br>0,44 ± 0,84	p = 0.8
**T0 – T2**	0,31 ± 0,69<br>0,41 ± 0,71	p = 1.00	0,13 ± 0,55<br>0,34 ± 0,65	p = 1.0
**T0 – T3**	0,31 ± 0,69<br>0,59 ± 0,95	p = 0.97	0,13 ± 0,55<br>0,38 ± 0,83	p = 1.0
**T1 – T2**	0,75 ± 0,95<br>0,41 ± 0,71	p = 0.44	0,44 ± 0,84<br>0,34 ± 0,65	p = 1.0
**T1 – T3**	0,75 ± 0,95<br>0,59 ± 0,95	p = 1.00	0,44 ± 0,84<br>0,38 ± 0,83	p = 1.0
**T2 – T3**	0,41 ± 0,71<br>0,59 ± 0,95	p = 1.00	0,34 ± 0,65<br>0,38 ± 0,83	p = 1.0
**Overall**	p = 0.002		p = 0.004	

T0 = baseline, T1 = after the first competitive video gaming session, T2 = after the ten-minute break, T3 = after the second competitive video gaming session.

Supplementary Table 3. Post-hoc analysis for Borg scale during the competitive video gaming sessions every 15 minutes.

	T0-15	T0-30	T0-45	T0-60	T0-75	T0-90	T2-15	T2-30	T2-45	T2-60	T2-75	T2-90
**T0-30**	1.000	-	-	-	-	-	-	-	-	-	-	-
**T0-45**	1.000	1.000	-	-	-	-	-	-	-	-	-	-
**T0-60**	0.265	0.566	0.695	-	-	-	-	-	-	-	-	-
**T0-75**	**0.006**	**0.016**	**0.022**	1.000	-	-	-	-	-	-	-	-
**T0-90**	**0.029**	0.081	0.104	1.000	1.000	-	-	-	-	-	-	-
**T2-15**	1.000	1.000	1.000	1.000	0.081	0.297	-	-	-	-	-	-
**T2-30**	0.849	1.000	1.000	1.000	1.000	1.000	1.000	-	-	-	-	-
**T2-45**	0.063	0.150	0.188	1.000	1.000	1.000	0.566	1.000	-	-	-	-
**T2-60**	**<0.001**	**0.001**	**0.001**	1.000	1.000	1.000	**0.006**	1.000	1.000	-	-	-
**T2-75**	**0.019**	0.055	0.071	1.000	1.000	1.000	0.211	1.000	1.000	1.000	-	-
**T2-90**	**<0.001**	**0.001**	**0.001**	1.000	1.000	1.000	**0.005**	1.000	1.000	1.000	1.000	-

T0-min = first competitive video gaming session, T2-min = second competitive video gaming session
Figures in bold indicate a significant value of at least p = 0.05

3 Supplementary Materials

Adjective list for assessing Perceived Physical State (PEPS)

(self-translated, non-validated version)

Introduction:

Please estimate, without much thought, the extent to which the following statements apply to your **current physical state** by placing a cross in the appropriate place.

**At the moment,** I feel physically...	**not at all**					**completely**
vigorous	0	1	2	3	4	5
energy less	0	1	2	3	4	5
immobile	0	1	2	3	4	5
exhausted	0	1	2	3	4	5
damaged	0	1	2	3	4	5
limber	0	1	2	3	4	5
drained	0	1	2	3	4	5
sick	0	1	2	3	4	5
flabby	0	1	2	3	4	5
strong	0	1	2	3	4	5
stiff	0	1	2	3	4	5
fit	0	1	2	3	4	5
limp	0	1	2	3	4	5
trained	0	1	2	3	4	5
weakened	0	1	2	3	4	5
healthy	0	1	2	3	4	5
stretchable	0	1	2	3	4	5
powerful	0	1	2	3	4	5
injured	0	1	2	3	4	5
flexible	0	1	2	3	4	5

**At the moment**, I have physical...	**not at all**					**completely**
pain	0	1	2	3	4	5
discomfort	0	1	2	3	4	5

Source:

Kleinert J. Adjektivliste zur Erfassung der Wahrgenommenen Körperlichen Verfassung (WKV). *Zeitschrift für Sportpsychologie* (2006) **13**:156–64. doi:10.1026/1612-5010.13.4.156
